# AI and Internet of Things for Chronic Obstructive Pulmonary Disease Remote Monitoring: Systematic Review of Exacerbation Prediction and Key Physiological Variables

**DOI:** 10.2196/84814

**Published:** 2026-05-06

**Authors:** Martina Montenegro, Jasper Gielen, Chunzhuo Wang, Bart Vanrumste, David Ruttens, Ruben Knevels, Jean-Marie Aerts

**Affiliations:** 1 Department of Biosystems, M3-BIORES, Division Animal and Human Health Engineering, KU Leuven, Castle Park Arenberg 30, Leuven, Belgium, 32 16321434; 2 Department of Electrical Engineering, e-Media Research Lab, Division STADIUS, KU Leuven, Leuven, Belgium; 3 Department of Respiratory Medicine, Ziekenhuis Oost-Limburg, Genk, Belgium; 4 Faculty of Medicine and Life Sciences, Hasselt University, Diepenbeek, Belgium; 5 Limburg Clinical Research Center/Mobile Health Unit, Faculty of Medicine and Life Sciences, Hasselt University, Hasselt, Belgium; 6 Future Health Department, Ziekenhuis Oost-Limburg, Genk, Belgium

**Keywords:** ECOPD, remote monitoring, machine learning, prediction, health care management, ML, AI, IoT, exacerbations of chronic obstructive pulmonary disease, artificial intelligence, Internet of Things

## Abstract

**Background:**

Chronic obstructive pulmonary disease (COPD) is a leading cause of morbidity and mortality worldwide, with frequent exacerbations of COPD (ECOPD) significantly impacting patient health and health care systems. Predicting ECOPD early would increase patients’ quality of life and decrease the economic burden. The advancement of wearable technologies and Internet of Things (IoT) sensors has enabled continuous remote monitoring (RM), offering new opportunities for early ECOPD prediction. However, effectively leveraging wearable data requires robust artificial intelligence (AI) frameworks capable of processing heterogeneous physiological and environmental information.

**Objective:**

This systematic review aims to provide a comprehensive overview of both hardware and software solutions for predicting ECOPD using RM. From the reviewed literature, we first focus on key physiological and environmental variables essential for COPD monitoring that can be extracted from wearables and IoT sensors. Second, we describe the wearable and IoT devices currently deployed in COPD management. Finally, we review machine learning, including deep learning models, used for ECOPD prediction, discussing limitations for real-world implementation. By bridging AI-driven data processing with real-world sensor applications, this review aims to outline the current landscape, existing challenges, and future directions for developing effective RM solutions for ECOPD predictions.

**Methods:**

A comprehensive search was conducted following the PRISMA (Preferred Reporting Items for Systematic Reviews and Meta-Analyses) guidelines to identify studies using AI or machine learning techniques for predicting ECOPD in in-home contexts.

**Results:**

This review identified 26 studies that met the inclusion criteria. Twenty studies aimed at predicting or detecting exacerbations at the onset. The variables tracked most frequently were heart rate (n=9), peripheral oxygen saturation (n=9), and symptoms (n=8). Daily or weekly sampling was most common (n=14). Most studies (n=13) applied machine learning models—primarily random forest (n=5), CatBoost (n=2), decision trees (n=2), and support vector machines (n=2). Deep learning was used in 3 papers, while the remaining applied rule-based logics and probabilistic models. Wearables and IoT were used in only 6 out of 20 studies. Six papers analyzed changes in vital parameters during prodromal phases, defined as the period shortly before the onset of an exacerbation. Three studies collected data continuously, 2 daily, and 1 compared once-daily versus overnight monitoring; 4 of these 6 used wearable devices.

**Conclusions:**

Overall, current evidence highlights the potential of continuous monitoring of physiological and environmental variables for early ECOPD prediction, offering advantages over questionnaires or once-daily measurements. While wearables and IoT devices show promise, their use remains limited. Many studies rely on balanced datasets that do not mirror real-world exacerbation patterns and lack external validation across diverse populations. Future research should emphasize large-scale validation, integration of multimodal data, and translation of AI models into clinically feasible tools to enable timely intervention and improve COPD management.

## Introduction

Chronic obstructive pulmonary disease (COPD) is a heterogeneous lung condition characterized by chronic respiratory problems, including dyspnea, cough, sputum production, and exacerbations. These issues can be attributed to abnormalities of the airways (bronchitis and bronchiolitis) and/or alveoli (emphysema) leading to persistent, often progressive, airflow obstruction [1]. COPD is a major global health issue, causing significant morbidity and mortality worldwide, and placing an increasing burden on health care systems and the economy. The current global estimated prevalence of 10.3% is projected to rise, particularly in high-income countries, mainly due to an aging population [1]. According to the World Health Organization, COPD was responsible for 3.5 million deaths in 2021 alone, making it the fourth leading cause of death worldwide [2].

The primary factors contributing to disease progression, hospitalization, and readmission are exacerbations of COPD (ECOPD), which refers to acute episodes of disease worsening. According to the Global Initiative for Chronic Obstructive Lung Disease (GOLD) definition, these are defined as “an event characterized by increased dyspnea and/or cough and sputum that worsens in less than 14 days, which may be accompanied by tachypnea and/or tachycardia and is often associated with increased local and systemic inflammation caused by infection, pollution, or other insults to the airways” [1,3]. ECOPD episodes are classified as mild or moderate, according to the medication treatment, or severe, if hospitalization or emergency room (ER) visits are required [1]. Thus, the severity of ECOPD is graded post hoc, based on the medication used and the setting, and is determined by the patient’s subjective perception of symptoms.

Currently, there is no single universally recognized gold standard to assess ECOPD. Instead, exacerbations are commonly identified through a combination of symptom-based or event-based assessments, patient self-reports, and clinical evaluation [4]. ECOPD is defined as event-based, using objective criteria such as health care use or specific treatments, including self-administration of medication or unscheduled visits to the ER and/or hospital admission. Alternatively, it can be defined as symptom-based, relying on patient-reported changes in symptoms. An increase in symptoms such as dyspnea, cough, or sputum production for a minimum number of consecutive days, or changes in symptoms recorded in daily diaries or questionnaires. However, it is logical that the symptoms precede the event, as they are the underlying cause.

It is estimated that approximately 30% to 50% of people diagnosed with COPD experience at least one exacerbation yearly, often requiring hospitalization to stabilize the patient [5]. A significant concern following these hospitalizations is the high rate of readmissions. Data from the European COPD audit revealed that 35.1% (5337/15,191) of patients were readmitted within 90 days of discharge [6]. ER visits and hospitalizations due to signs and symptoms of ECOPD are the main reason for COPD-related costs, which can account for up to 56% of the annual respiratory disease budget, equating to €38.6 billion (US $45.7 billion) [7,8]. A qualitative study by Locke et al [9] found that most patients can distinguish worsening disease symptoms from baseline symptoms, but a reluctance to seek care was a typical response of many persons with COPD when developing ECOPD. Most delayed action until a critical threshold was reached, when the urgency of their symptoms surpassed the personal and initial barriers they faced. In contrast, early recognition and prompt treatment by a physician can improve the outcomes of ECOPD [10]. These findings underscore the importance of implementing strategies aimed at early recognition of disease worsening, enabling prompt therapeutic action.

To achieve effective early prediction, it is essential to identify key parameters for monitoring and establish consensus on alerting thresholds. This process involves identifying patients at high risk before the onset of severe symptoms. Machine learning (ML) models, particularly those integrated with emerging Internet of Things (IoT) technologies, offer promising solutions for accurately and remotely sensing vital parameters. Equally important is determining the optimal time window for early prediction. This window should allow for sufficient lead time to initiate interventions that could prevent or attenuate an exacerbation. If the time frame is too short, there may not be sufficient time to act effectively. Conversely, a time frame that is too long, such as forecasting ECOPD risk over the next 12 months, may have limited clinical utility, as it does not inform the timing of specific interventions. Therefore, determining the optimal lead time for prediction is critical for maximizing both predictive accuracy and clinical actionability. This choice must balance the need for early warning with the relevance and reliability of available data in the lead-up to an exacerbation.

Beyond achieving accurate early prediction, ensuring clinical translation and adoption is equally important. Recent reviews, such as Qi et al [11], underscore the importance of structured validation frameworks and clinical interpretability as essential prerequisites for integration into clinical practice. Consequently, these considerations are as important as the previously discussed factors in the development of early prediction models. In particular, it is essential to ensure that the model is both generalizable and explainable for successful clinical adoption.

**Figure 1.**
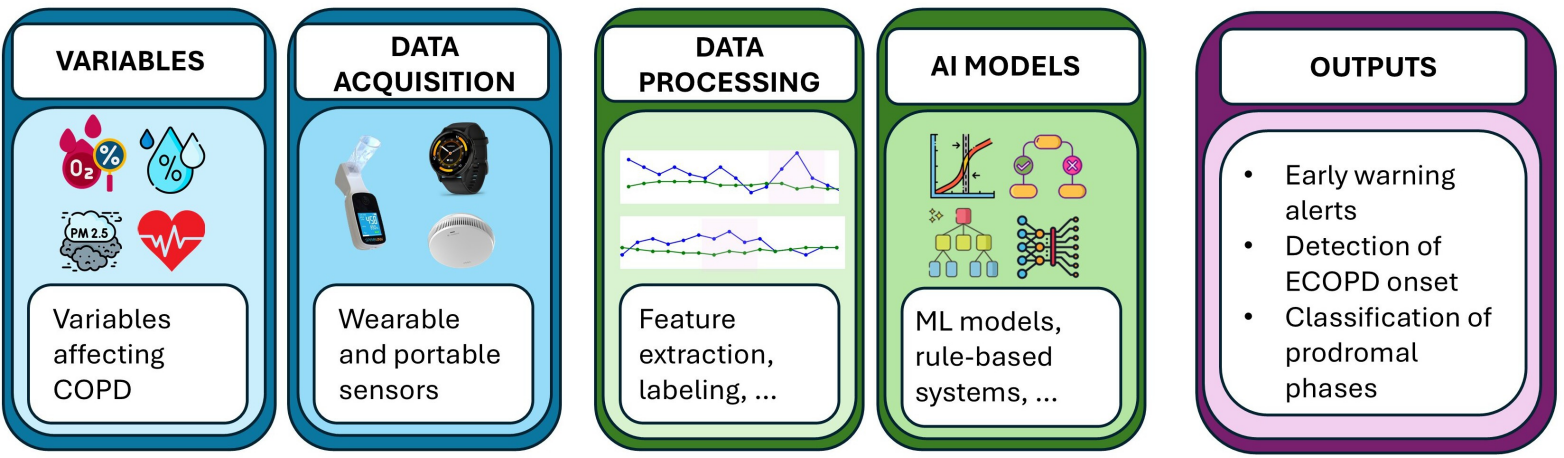
Schematic overview of exacerbations of chronic obstructive pulmonary disease prediction pipeline. AI: artificial intelligence; COPD: chronic obstructive pulmonary disease; ECOPD: exacerbations of chronic obstructive pulmonary disease.

From a practical perspective, ECOPD prediction involves several fundamental steps, including the identification of physiological variables and external factors that change and/or contribute to the lead-up to an exacerbation. It also involves the selection of wearable and/or portable devices to collect these variables. It is then important to identify key parameters that change during prodromal phases, that is, before exacerbation of symptoms, as such information may positively influence model performance. The collected data are then preprocessed using various techniques and subsequently fed into artificial intelligence (AI)/ML algorithms. The objective of the algorithm could be early warning generation (with varying prediction windows), the detection of exacerbation onset, or the classification of prodromal phases. The entire ECOPD prediction pipeline is illustrated in Figure 1.

The goal of this systematic review is to analyze existing research on AI- and IoT-based wearable systems for remote COPD monitoring, focusing on which variables are evaluated to assess and/or predict exacerbations, the granularity of the collected data, and the device type used to enable remote patient monitoring (RPM). It highlights state-of-the-art results on prediction accuracy and area under the curve (AUC), outlining which AI and ML techniques are most commonly used. It focuses on examining existing data frameworks, the AI models used for predicting ECOPD, and identifying challenges in their real-world application, as well as gaps in the current research.

The research questions can be summarized as follows:

1. Which key physiological variables are most relevant for predicting COPD exacerbations? Does the data resolution (eg, sampling frequency) influence model performance? Is there any standardization in the IoT devices used for RPM in COPD management?
2. Which AI models are used to predict ECOPD, and how accurate are they?
3. What are the gaps in current research regarding wearable IoT and AI for COPD monitoring, and what should be prioritized in future studies?

The first research question aims to identify which physiological and environmental variables are most commonly used, exploring the frequency of data collection and its impact on the outcome. It further reviews the types of wearables and IoT devices used, and whether there is any standardization across studies. The second research question discusses the ML techniques applied, including performance metrics and limitations. It also clarifies how exacerbations are defined, which influences model training and evaluation. Finally, the third one synthesizes insights from all thematic areas to identify limitations in current approaches and propose future directions. Each of these points is examined thoroughly in the “Discussion” section.

To support this analysis, we also categorize the reviewed articles based on their primary objectives. Studies that focused on developing models for predicting exacerbations are discussed in the section titled “Exacerbation Prediction,” while those that examined changes in vital parameters preceding exacerbations are presented in the section titled “Variable Changes in Prodromal Phases.” This second group of papers was deliberately included to highlight the importance of identifying discriminative input variables, which is critical for improving model performance and capturing the complex patterns of disease progression. Despite this thematic separation, the discussion of results remains structured around the 3 central research questions, ensuring a coherent and focused analysis.

Among the literature on reviewing ECOPD, this review adds key insights for developing an effective remote system for early ECOPD prediction. We identify monitored variables that have discriminative power to predict ECOPD, underscore the need for a universal definition of ECOPD, and review state-of-the-art telemonitoring solutions and early prediction methods. By doing so, this review aims to lay a solid foundation for improving outcomes and their validation within the context of RPM for ECOPD. This review aims to emphasize the necessity of future research in 3 key areas: reducing the burden on medical specialists through automated data detection and prediction, alleviating patient burden by minimizing active participation in data collection, and enhancing predictive models through multimodal strategies.

## Methods

### Eligibility Criteria, Information Sources, and Search Strategy

The review was conducted in accordance with the PRISMA (Preferred Reporting Items for Systematic Reviews and Meta-Analyses) guidelines [12]. A comprehensive search was carried out in PubMed, Scopus, IEEE Xplore, and Embase during March 2025. The search strings can be found in Multimedia Appendix 1.

Only peer-reviewed, full-text articles published in English were considered. The following publication types were excluded: systematic reviews, meta-analyses, book chapters, editorials, letters, and conference abstracts. However, full papers in conference proceedings were included.

Studies were eligible for inclusion if they met the following criteria:

1. Population: patients with COPD, undergoing exacerbations of the condition.
2. Settings: RPM
3. Measurements: objective (ie, measurable) physiological variables

The search strategy was based on three core concepts:

1. Wearable and remote monitoring (RM) technologies (eg, wearable sensors, smartwatches, home monitoring, and IoT sensors)
2. COPD
3. ML and AI approaches for the prediction or detection of ECOPD

Both controlled vocabulary terms (eg, MeSH [Medical Subject Headings] in PubMed) and free-text keywords were used. The search strategies were adapted for each database’s specific syntax and indexing system.

No additional filters or limitations were applied during the search process.

### Selection Process and Data Collection

The screening process was conducted using Rayyan (Qatar Computing Research Institute) [13], a web-based tool for systematic review management. Duplicate records were first identified automatically by the platform, but their final removal was performed manually by the first reviewer to ensure accuracy.

Two reviewers (MM and JG) independently screened the articles, initially based on titles and abstracts, followed by full-text screening for eligibility. In cases of disagreement, conflicts were resolved through discussion until consensus was reached. Articles were selected for inclusion if they met all predefined eligibility criteria.

Following the screening, the first reviewer (MM) manually extracted the relevant data using Microsoft Excel. The second reviewer (JG) subsequently verified the data for completeness and accuracy.

The following data items were extracted: title, authors, year of publication, journal, aim of the study, population characteristics, study duration, definition of exacerbation, monitored parameters, devices used, sampling frequency, machine learning or deep learning models used, and main outcomes.

### Risk of Bias Assessment

The risk of bias was assessed by one reviewer (MM) and verified by a second (JG). Any disagreements between reviewers were resolved through discussion to reach consensus. To ensure a comprehensive evaluation, several risk of bias assessment tools were applied. Twenty prediction model studies were evaluated using the PROBAST (Prediction Model Risk of Bias Assessment Tool) [14], which is specifically designed for evaluating prediction model studies. PROBAST assesses bias across 4 domains: participants, predictors, outcomes, and analysis. Five observational studies were evaluated using the Newcastle-Ottawa Scale [15], while the only randomized controlled trial (RCT) intervention study was assessed with RoB 2 (Cochrane Collaboration) [16].

## Results

### Study Selection

**Figure 2.**
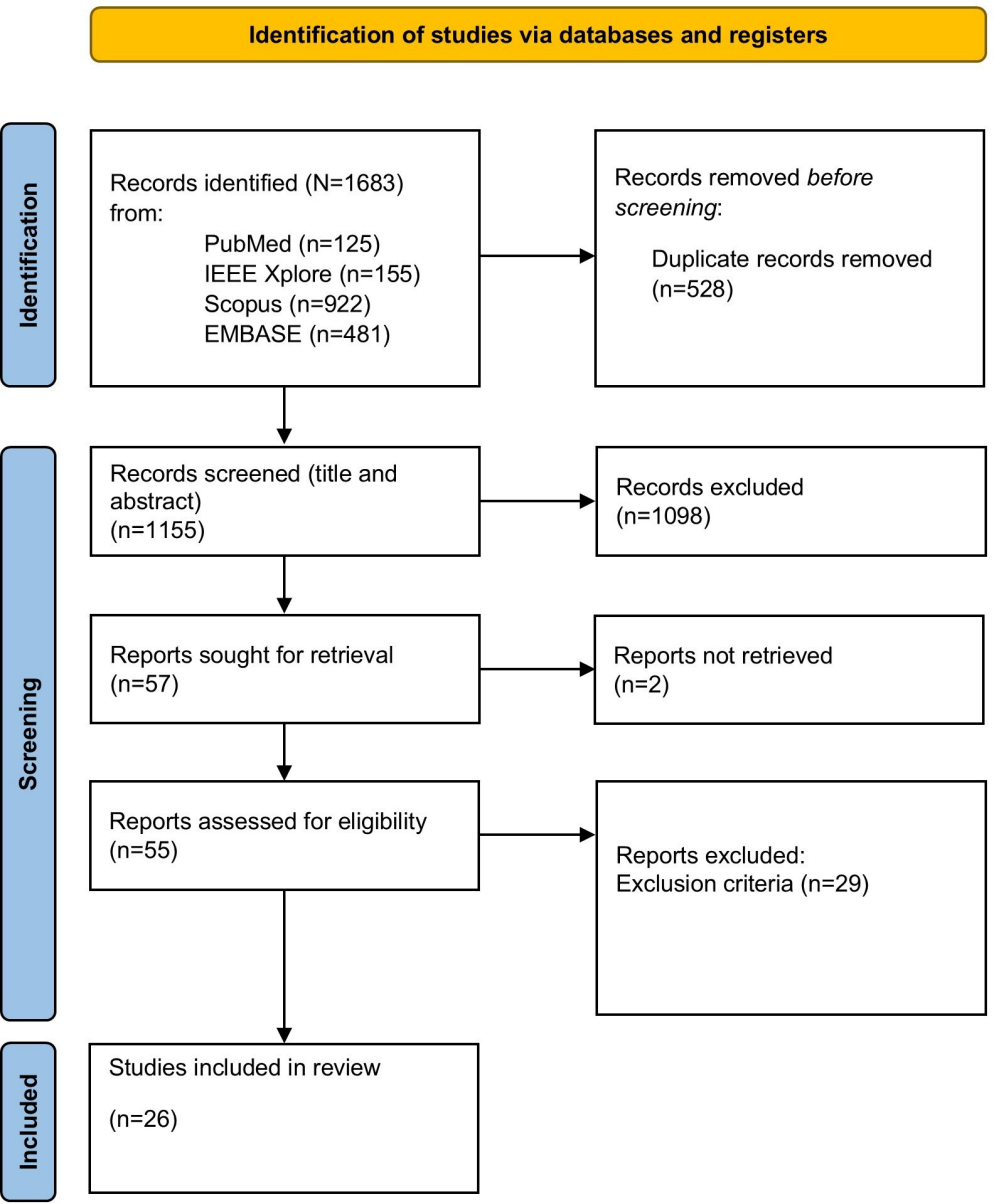
PRISMA (Preferred Reporting Items for Systematic Reviews and Meta-Analyses) flow diagram.

The study selection process is illustrated in the PRISMA flow diagram (Figure 2).

The systematic search yielded a total of 1683 records: 125 from PubMed, 155 from IEEE Xplore, 922 from Scopus, and 481 from Embase. Prior to screening, 528 duplicate records were removed, resulting in 1155 unique records for title and abstract screening. Based on the predefined inclusion and exclusion criteria, 1098 records were excluded at this stage.

The remaining 57 articles were retrieved for full-text assessment. Of these, 2 records could not be accessed despite efforts to contact the authors via ResearchGate. As a result, 55 full-text articles were assessed for eligibility.

Full-text assessment followed the same inclusion and exclusion criteria as title and abstract screening. After full-text screening, 26 studies [17-42] met the inclusion criteria and were included in this systematic review.

### Study Characteristics

#### Exacerbation Prediction

The key characteristics of the studies related to exacerbation prediction are summarized in Table 1. Across the 20 included studies [17-36], sample sizes range from 9 to 177 participants, and study durations vary from 1 month to 4 years. Some studies are outliers. For example, Wu et al [17] do not explicitly state the length of the monitoring period, and in the study by Lin et al [18], the follow-up of the participants ranged from 16 to 448 days, with an average testing period of 206 days per patient.

Definitions of exacerbation also varied substantially. In 3 studies [20,28,35], exacerbations were identified by clinical judgment; 4 studies [24,25,27,29] used event-based definitions such as hospitalization or changes in medication. Two studies relied on symptom-based criteria [23,31], 2 adopted the GOLD definition [19,22], and one relied on self-reported exacerbations, validated by clinicians [21]. In the study by Wu et al [17], the definition was not clearly reported (“NA”), but it is highly probable that they adopted the same of their previous study [19]. In other studies, authors used custom rule-based criteria, often operationalized in the study’s algorithm.

**Table 1.** Characteristics of included studies (exacerbation prediction).

Study (year)	Population (study duration)	Exacerbation definition	Model	Parameters monitored and sampling
Wu et al [19] (2021)	67 (4 months)	GOLD^a^ guidelines	ML^b^ and DNN^c^	Symptoms - activity, HR^d^, Env^e^/daily - continuous
Merone et al [20] (2017)	22 (6 months)	Clinicians	Binary FSM^f^	SpO_2_^g^, HR/3 times a day
Atzeni et al [21] (2022)	101 (6 months)	Self-reported, clinicians validation	Clustering and ML	Symptoms, sleep quality, and PEF^h^, EHR^i^ - Env/daily - continuous
Yin et al [22] (2024)	66 (6 months)	GOLD guidelines	ML	Respiratory variables, breathing sounds/2 times a day
Fernandez-Granero et al [23] (2018)	15 (6 months)	Symptom-based	Decision Tree Forest	Breathing sounds/daily
Wu et al [17] (2022)	177 (NA^j^)	NA	ML and DNN	Symptoms - activity, HR, Env/daily - continuous
Kronborg et al [24] (2021)	9 (2 years)	Event-based	ML	SpO_2_, HR, BP^k^/daily to weekly
Fernandez-Granero et al [25] (2015)	15 (6 months)	Event-based	PCA-SVM^l^	Breathing sounds/daily
Nunavath et al [26] (2018)	94 (2 years)	Medical rule	LSTM^m^	Symptoms, HR, SpO_2_/daily
Crooks et al [27] (2021)	28 (90 days)	Event-based	XGB^n^ and rule-based system	Cough monitoring/continuous at the bedside
Kolozali et al [28] (2023)	106 (6 months)	Clinicians	PLCA-LDS-3D^o^	Audio and PEF - Env, accelerometer/daily - continuous
Shah et al [29] (2017)	110 (1 year)	Event-based	Short-period trend analysis and LR^p^	HR, SpO_2_, RR^q^/daily to weekly
Orchard et al [30] (2018)	135 (1 year)	Hospitalization	Neural Net	Symptoms, medications, HR, SpO_2_, outdoor weather/daily
Van der Heijden et al [31] (2014)	10 (4 weeks)	Symptom-based	Temporal Bayesian Network	Symptoms, FEV1, SpO_2_/daily
Mohktar et al [32] (2015)	21 (1 year)	Rule-based	CART^r^	FEV1^s^, SpO_2_, RR, HR, T^t^, weight/daily
Jin et al [33] (2018)	22 (1 month)	Hospitalization	ML	Respiratory variables, SpO_2_/4 hours a day
Lin et al [18] (2020)	16 (2 weeks-1 year)	ER^u^ visits	Trend analysis and rule-based system	Activity, sleep/continuous
Moraza et al [34] (2025)	149 (4 years)	Rule-based	CatBoost and DL^v^	Symptoms, HR, RR, SpO_2_, steps, T/daily
Patel et al [35] (2021)	90 (6 months)	Clinicians	Decision Tree	Symptoms - FEV1 - CRP^w^ daily - weekly - at alerting thresholds
Hsiao et al [36] (2023)	13 (6 months)	Rule-based	RF^x^	HRV^y^/3 times a day

a GOLD: Global Initiative for Chronic Obstructive Lung Disease.

b ML: machine learning.

c DNN: deep neural network.

d HR: heart rate.

e Env: environmental variables.

f FSM: Finite State Machine.

g SpO2: peripheral capillary oxygen saturation.

h PEF: peak expiratory flow.

i EHR: electronic health records.

j Not available.

k BP: blood pressure.

l PCA-SVM: principal component analysis – support vector machine.

m LSTM: Long Short-Term Memory.

n XGB: Extreme Gradient Boosting.

o PLCA-LDS-3D: Probabilistic Latent Component Analysis – Linear Dynamical System in 3D.

p LR: logistic regression.

q RR: respiratory rate.

r CART: Classification and Regression Trees.

s FEV1: Forced Expiratory Volume in 1 second.

t T: temperature.

u ER: emergency room.

v DL: deep learning.

w CRP: C-reactive protein.

x RF: random forest.

y HRV: heart rate variability.

Most of the included studies used ML techniques, with a few also incorporating deep learning models. Notably, time-series analysis was explicitly used in only a subset of the studies, whereas many applied ML techniques to static or aggregated data. Of the reviewed studies, 13 reported their best performance using traditional ML techniques [17,21-25,28,29,32-36], while 2 relied on rule-based systems [18,27], 1 on a Bayesian model [31], 1 on a finite state machine [20], and 3 on deep learning architectures [19,26,30]. Overall, the prevalence of simpler modeling approaches is consistent with the generally small size of the datasets used in these studies.

The monitored variables and sampling frequencies exhibit significant heterogeneity in terms of both sensor modalities and data collection protocols. The variables tracked most frequently are heart rate (HR; 9 studies [17,19,21,24,26,29,30,32,34]), peripheral capillary oxygen saturation (SpO2; 9 studies [17,20,24,26,29,30,32-34]), and symptoms (8 studies [17,19,21,26,30,31,34,35]).

To clarify which parameters are being monitored in each study, we have categorized the papers according to the physiological and environmental variables they focus on (1) monitoring of physiological variables (eg, oxygen saturation and HR) [18,20,24,26,29,31,32,34,36], (2) monitoring of respiratory variables (eg, forced vital capacity [FVC] and peak expiratory flow [PEF]), breathing sounds, or coughing [22,23,25,27,33], (3) monitoring of environmental variables [21,28], (4) monitoring of both physiological and environmental variables [17,19,30], and (5) monitoring also forced expiratory volume in 1 second (FEV1) [21,28,31,32].

The only exceptions stand in Patel et al [35], which is monitoring FEV1 and finger-prick C-reactive protein, where the latter is a physiological variable but invasively measured from blood samples.

The sampling frequency varied across studies. Daily sampling was used in 7 studies [17,20,23,24,26,30,32], while a few studies collected measurements multiple times per day (2‐3 times). Some studies collected data weekly, and 6 studies [17,21,27,28,30,34] used continuous monitoring. Only in the study by Lin et al [18], the sampling frequency explicitly stated, set at 20 Hz. For the remaining studies, no raw sampling is specified. Specifically, in Wu et al [17,19], data synchronization occurs every 15 minutes, while in the study by Atzeni et al [21], it happens every 30 hours. Once again, in the study of Crooks et al [27], the system continuously analyzes audio input, but the actual sampling frequency of the raw audio is not specified. In the study by Kolozali et al [28], the sampling frequency is not explicitly reported. However, based on the data volume (54 million points over 106 participants), acquisition intervals likely range from subminute to several minutes depending on the sensor.

As the review focuses on wearables and IoT sensors, it is important to note that only a minority of the studies explicitly mention the specific devices used, and that there is substantial heterogeneity in device types, specifically wearable and portable sensors.

Wearable devices, which are continuously worn on the body, included the Fitbit Versa smartwatch and EDIMAX AirBox environmental sensor [17,19], custom-made Personal Ambient Monitor worn around the waist [21], a stationary microphone (Jabra 510+, Jabra GN) at the bed table [27], air pollution sensors and activity-related sensors, not further specified [28], accelerometer-based wrist-worn wearable devices: GeneActiv [18], O2 of Zoetek Inc smartwatch [36].

Portable devices, which are intermittently used or carried by the patient, included pulse oximeter ([20,29,34] which included also a pedometer), portable spirometers and electronic stethoscopes [22], electronic sensor ad hoc designated [23], electronic stethoscopes and a microphone [25], home monitoring unit (TeleMed-Care Health Monitor: TMC-Home, TeleMedCare Pty Ltd) [32], noninvasive ventilator (RESmart GII BPAP Y-25T, BMC Medical Co, Ltd) [33], and Vitalograph, Ireland, to measure FEV1 [35].

This taxonomy highlights the lack of standardization in both device selection and reporting across studies, complicating direct comparison and synthesis of findings.

#### Variables Changes in Prodromal Phases

The key characteristics of the studies related to the analysis of physiological variables in prodromal phases are presented in Table 2.

The 6 included studies [37-42] report sample sizes ranging from 16 to 104 participants, with durations spanning 21 days to 6 months. Two studies stand out as outliers: in Kim et al [42], it is not explicitly stated, and in Cooper et al [41], all participants collectively provided 2618 days.

The definitions of exacerbation also varied. Two studies used event-based definitions [39,40], 2 studies relied on symptom-based criteria [37,38], and one adopted multiple definitions [41], in order to see how many different exacerbations were captured, according to the applied definition. In one instance, the definition was not clearly reported (“NA”), not even in the study protocol [43].

**Table 2.** Characteristics of included studies (variable changes in prodromal phases).

Study	Population (study duration)	Exacerbation definition	Model	Parameters monitored and sampling
Hawthorne et al [37] (2022)	35 (6 weeks)	Symptom-based (mild), symptom-based and medication (moderate), hospitalization (severe)	NA^a^	RR^b^, HR^c^, T^d^, activity/continuous
Coutu et al [38] (2024)	21 (21 days)	Symptom-based	Linear mixed- effects models	RR, RRV^e^, HR, HRV^f^, T, ED^g^ activity, activity, sleep/continuous
Zimmerman et al [39] (2020)	16 (8‐9 months	Increase in respiratory symptoms requiring oral corticosteroids and/or antibiotics, with or without medical review and/or hospitalization.	Linear mixed- effects models	Respiratory variables/daily
Al Rajeh et al [40] (2020)	83 (6 months)	Event-based	NA	Symptoms - PEF^h^, HR, SpO_2_^i^/daily- daily vs overnight
Cooper et al [41] (2020)	17 (2618 patient-days)	Multiple	SPC^j^, concordance analysis	Symptoms, respiratory variables, SpO_2_/daily
Kim et al [42] (2021)	104 (NA)	NA	LR^k^	PM2.5^l^/continuous

a Not available.

b RR: respiratory rate.

c HR: heart rate.

d T: temperature.

e RRV: respiratory rate variability.

f HRV: heart rate variability.

g ED: electrodermal activity.

h PEF: peak expiratory flow.

i SPO2: peripheral capillary oxygen saturation.

j SPC: statistical process control.

k LR: logistic regression.

l PM2.5: particulate matter ≤2.5 µm.

The included studies used statistical analysis to inspect the correlations between exacerbations and variation in physiological variables, including HR, respiratory variables, and in one study [42], PM2.5 (particulate matter ≤2.5 µm) is considered. Three studies collect data continuously [37,38,42], 2 daily [39,41], and one assesses the difference between once daily and overnight monitoring [40].

Regarding the devices used, it can be highlighted that 4 studies [37,38,40,42] out of 6 [37-42] use wearable devices, specifically:

1. Hawthorne et al [37]: chest-worn multiparameter monitoring system (Equivital LifeMonitor EQ02)
2. Coutu et al [38]: a biometric wristband (EmbracePlus, Empatica Inc) and a biometric ring (Oura Gen III, Oura Health Oy)
3. Al Rajeh et al [40]: wristband pulse oximeter (Nonin 3150) for overnight monitoring (measurement recorded every 4 s), or finger pulse oximeter (Nonin G92) for once-daily (morning) monitoring.
4. Kim et al [42]: IoT to measure PM2.5 indoor: a sensor-based light scattering measurement device (CP-16-A5, Aircok Inc).

Meanwhile, Zimmermann et al [39] and Cooper et al [41] both use portable devices to monitor respiratory trends daily. Specifically, the former uses a FOT (Forced Oscillation Technique) telemonitoring device (Resmon Pro Diary, Restech Srl) to monitor respiratory variables every morning, and the latter RPM (handheld spirometer [SpiroPro, eResearch Technology] and pulse oximeter [Onyx II, Nonin Medical]).

#### Risk of Bias

The only RCT [40] has been assessed as low risk in the randomization process, measurement of the outcome, and selection of the reported results. However, certain issues have been identified regarding deviations from the established interventions. These include the unfeasibility of binding the participants and a high rate of participant dropout. Due to these concerns, a high risk has been assigned to missing data.

The Newcastle-Ottawa Scale results are reported in Table 3. Overall, no study raises concerns.

**Table 3.** Risk of bias assessment (Newcastle-Ottawa Scale).

Study (year)	Selection (0‐4)	Comparability (0‐2)	Outcome (0‐3)	Total score (0‐9)
Hawthorne et al [37] (2022)	3	2	3	8/9
Coutu et al [38] (2024)	3	2	3	8/9
Zimmerman et al [39] (2020)	3	2	3	8/9
Cooper et al [41] (2020)	3	2	3	8/9
Kim et al [42] (2021)	3	2	2	7/9

Finally, most of the studies are related to prediction models; therefore, the risk of bias has been assessed with the PROBAST, which evaluates Participants and Data Sources (P), Predictors (Pr), Outcome (O), and Analysis (A). The results are summarized in Table 4. A high risk of bias was identified in the study by Wu et al [17] due to an unclear definition of outcomes and ambiguity in the sample breakdown, which adds uncertainty to the findings. In the study by Kronborg et al [24], the use of a very small cohort (9 patients selected from 108), a low number of positive samples (17 exacerbation periods), and reliance on self-reported data introduce a high risk of bias. Nunavath et al [26] present potential bias arising from synthetic oversampling, exclusion of one class, and the use of triage labels as proxies for exacerbation severity. Kolozali et al [28] apply complex modeling techniques without external validation and with unclear measures to control for overfitting, raising concerns about model reliability. In the study by van der Heijden et al [31], the small sample size, the use of self-reported predictors that are not clearly linked to outcomes, and the use of symptoms to both define exacerbations and as input features for prediction models contribute to a heightened risk of bias.

In the “Discussion” section, we discuss how biases in study design and analysis can lead to inflated predictive performance metrics.

**Table 4.** Risk of bias assessment (PROBAST^a^).

Study (year)	Participants	Predictors	Outcome	Analysis
Wu et al [19] (2021)	Low	Low	Low	Some concerns
Merone et al [20] (2017)	Low	Low	Low	Some concerns
Atzeni et al [21] (2022)	Low	Low	Low	Low
Yin et al [22] (2024)	Low	Low	Unclear	Low
Fernandez-Granero et al [23] (2018)	Low	Low	Low	Some concerns
Wu et al [17] (2022)	Low	Low	High	Unclear
Kronborg et al [24] (2021)	High	Low	Some concerns	High
Fernandez-Granero et al [25] (2015)	Low	Low	Low	Some concerns
Nunavath et al [26] (2018)	High	Low	Some concerns	High
Crooks et al [27] (2021)	Low	Low	Low	Low
Kolozali et al [28] (2023)	Low	Low	Low	High
Shah et al [29] (2017)	Low	Low	Low	Low
Orchard et al [30] (2018)	Low	Low	Low	Low
Van der Heijden et al [31] (2014)	Low	Some concerns	High	Some concerns
Mohktar et al [32] (2015)	Low	Low	Low	Low
Jin et al [33] (2018)	Low	Low	Low	Some concerns
Lin et al [18] (2020)	Low	Low	Low	Some concerns
Moraza et al [34] (2025)	Low	Some concerns	Low	Some concerns
Patel et al [35] (2021)	Low	Low	Low	Low
Hsiao et al [36] (2023)	Low	Low	Low	Some concerns

a PROBCAST: Prediction model Risk of Bias Assessment Tool.

### Study Analyses

#### Exacerbation Prediction

The results of the studies related to exacerbation prediction are summarized in Table 5. As anticipated, a broad range of ML and rule-based models have been deployed, with most aiming to forecast exacerbation of COPD between 1 and 7 days ahead. Furthermore, several studies reported overall accuracy without sufficient sensitivity-specificity trade-offs, thereby limiting assessment of clinical utility, and a lack of standardized reporting metrics hinders cross-study comparability. For this reason, we focus our comparisons on studies reporting the same performance metrics.

**Table 5.** Results of included studies (exacerbation prediction).

Study (year)	Outcomes	Performance metrics	Notes
Wu et al [19] (2021)	Early detection of exacerbations (7 days)	Accuracy=0.9357, AUROC^a^=0.9699, sensitivity=0.9452, specificity=0.9253, precision=0.9393, *F*_1_-score=0.9323	DNN^b^ on balanced dataset+feature importance
Merone et al [20] (2017)	Detection of the onset of ECOPD^c^	Accuracy=98.4, recall=92.9, specificity=99.3, precision=95.1, *F*_1_-score=94.0	Participant specific
Atzeni et al [21] (2022)	Early detection of ECOPD (1 day)	Cluster 1: AUC^d^=0.90, AUPRC^e^=0.7, sensitivity=0.83, specificity=0.86; Cluster 2: AUC=0.82, AUPRC=0.56, sensitivity=0.75, specificity=0.78	KNN^f^+RF^g^ + SHAP^h^
Yin et al [22] (2024)	Predict ECOPD (k=1, 2, 3 days)	AUC (k=1)=0.9721 (95% CI 0.9623‐0.9810), *F*_1_-score=0.846	CatBoost, balanced test set+feature importance
Fernandez-Granero et al [23] (2018)	Early detection of ECOPD (4.4 days margin)	Accuracy=87.8%, sensitivity=78.1%, specificity=95.9%, PPV^i^=94.1%, NPV^j^=83.9%, *F*_1_-score=0.8	NA^k^
Wu et al [17] (2022)	Early detection of ECOPD (7 days)	Accuracy=72.4%, sensitivity=62.5%, specificity=78.3%, precision=63.3%, *F*_1_-score=62.9%	DNN, external test set, feature importance, and SHAP
Kronborg et al [24] (2021)	Classification of prodromal periods (14 days)	AUC=0.95, sensitivity=0.94 (AUC gain=0.24 in a 2-layer model)	SVM^l^ (RBF^m^), 2-layer model
Fernandez-Granero et al [25] (2015)	Early detection of ECOPD (mean 5, SD 1.9 days)	Accuracy=75.8%, sensitivity=73.76%, specificity=97.67%, PPV=84.66%, NPV=95.53%	NA
Nunavath et al [26] (2018)	ECOPD prediction (k=1, 3 days)	1-day: accuracy=82.5%, loss=0.50; 3-day: accuracy=88%, loss=0.345	Excludes moderate risk patients
Crooks et al [27] (2021)	Alarm system (3 days ahead)	Cough: alerts 3.4 (SD 2.8) days early (success rate=45%), 1 false/100 days; Questionnaire: alerts 3.4 (SD 2.9) days early (success rate: 88%), 1 false/10 days	Participant-specific baseline for cough frequency
Kolozali et al [28] (2023)	Prediction of daily symptoms (1 day in advance)	Accuracy=38.67% and *F*_1_-score=0.19 (personalized), 19% and 0.10 (population)	Includes recovery phase
Shah et al [29] (2017)	Early detection of ECOPD (7 days)	Mean AUC=0.682 (95% CI 0.681‐0.682), specificity=36% (at 80% sensitivity), 68% (at 60% sensitivity)	Analysis of prodromal variables
Orchard et al [30] (2018)	Next day hospital admission prediction	AUC=0.740 (95% CI 0.673‐0.803)	No improvement with weather data
Van der Heijden et al [31] (2014)	Early detection of ECOPD (1 day)	Dval^n^ AUC=0.90, TPR^o^=0.87, FPR^p^=0.12; Ddedup^q^ AUC=0.82, TPR=0.78, FPR=0.19	External validation cohort, Bootstrapping dataset
Mohktar et al [32] (2015)	Classify high or low risk (1-day advance)	Accuracy=71.8%, specificity=80.4%, sensitivity=61.1%, PPV=71.4%, NPV=72%	Stable days excluded, feature importance
Jin et al [33] (2018)	Classification of prodromal periods (7 days)	Accuracy=74.5% (LDA^r^), 73.7% (SVM), 75% (RF); sensitivity=NA, 77.6%, 78.3%; specificity: lowest, 42.9%, 42%	Feature importance
Lin et al [18] (2020)	30-day hospital readmission prediction	Sensitivity=62.96%, precision=37.78%, miss rate=37.04%, FDR^s^=62.22%	NA
Moraza et al [34] (2025)	Early detection of ECOPD (3 days)	Test AUROC=0.91, AUPRC=0.53; prospective: AUROC=0.89, AUPRC=0.56	SHAP analysis, prospective validation data
Patel et al [35] (2021)	Early detection of ECOPD (7, 3 days [median, IQR])	Sensitivity=97.9%, specificity=84%, PPV=38.4%, NPV=99.8%, accuracy=85.3%	Participant-specific baseline
Hsiao et al [36] (2023)	Early detection of ECOPD (7 days)	Accuracy=0.94%, precision=0.57%, sensitivity=0.48%, specificity=0.93%	Rule-based predictions

a AUROC: area under the receiver operating characteristic curve.

b DNN: deep neural network.

c ECOPD: exacerbation of chronic obstructive pulmonary disease.

d AUC: area under the curve.

e AUPRC: area under the precision-recall curve.

f KNN: k-nearest neighbors.

g RF: random forest.

h SHAP: Shapley Additive Explanations.

i PPV: positive predictive value.

j NPV: negative predictive value.

k NA: not available.

l SVM: support vector machine.

m RBF: radial basis function.

n Dval: validation dataset.

o TPR: True positive rate.

p FPR: false positive rate.

q Ddedup: deduplicated dataset.

r LDA: linear discriminant analysis,

s FDR: false discovery rate.

Deep neural networks and tree-based ensemble methods achieved strong performance with AUC values approaching 90%. Wu et al [19] reported one of the best-performing models using a deep neural network with a 7-day prediction window, achieving an area under the receiver operating characteristic curve (AUROC) of 0.97, accuracy of 93.6%, and sensitivity of 94.5%. Nonetheless, the test set is balanced 1:1, thus not representing the real-world distribution. Similarly, CatBoost-based models by Yin et al [22] and Moraza et al [34] achieved AUROC values above 0.89, indicating strong discriminatory power for short-term prediction. However, Yin et al [22] used a 5-fold cross-validation with a balanced test set, which does not reflect real-world class imbalance.

Other models, such as those by Atzeni et al [21] and Kronborg et al [24], also achieved high AUROC scores (≥0.90) in specific patient clusters or multilayer support vector machine setups.

Only Wu et al [17] and van der Heijden et al [31] performed external validation on a different cohort, while Moraza et al [34] used prospective validation data sampled from the sample cohort.

Studies using random forest models (eg, [17,33,36]) demonstrated varying levels of performance. Wu and Jin typically reported moderate overall accuracies between 72% and 80%, with trade-offs in sensitivity and specificity. Jin et al [33] reported relatively balanced sensitivity (~76%‐78%) but consistently low specificity (~41%‐43%), suggesting a higher false positive rate. In contrast, Hsiao et al [36] reported a notably high accuracy of 0.94 and specificity of 0.93, but with lower precision (0.57) and sensitivity (0.48), highlighting strong negative class identification at the cost of reduced detection of true positives.

Models with rule-based or alert systems (eg, [27]) demonstrated real-world feasibility with fewer false positives, though at the expense of lower sensitivity (45% ECOPD detection with cough-based alarms), also using baseline personalization. Personalization appeared to significantly improve predictive performance. Patel et al [35] used a personalized baseline approach, achieving high sensitivity (97.9%) and negative predictive value (99.8%), despite a modest positive predictive value (PPV; 38.4%), indicative of a low false-negative rate, which is important for safety but less efficient for resource use. Other studies have also underscored the value of patient-specific modeling (eg, [20] with 98.4% accuracy in a participant-specific setup).

Lower-performing models (eg, [28]) demonstrated limited predictive power in both personalized (38.7%) and population-level (19%) settings, potentially due to inclusion of recovery phases or inadequate feature representation. Similarly, Lin et al [18] achieved moderate sensitivity (62.9%) but low precision (~38%), leading to high false discovery rates.

Finally, 3 studies [23,25,26] highlighted temporal margins for predictions: they reported early detection windows of ~3‐5 days with variable sensitivity and precision, balancing warning time with model reliability. Notably, Nunavath et al [26] excluded deteriorating patients from their model, considering only stable and urgent cases—once again failing to reflect real-world distributions.

Studies can be further categorized according to the selection of the prediction window [17,19,29,35,36]. Predict exacerbations 7 days ahead. Choosing a shorter time window (1‐3 days), there are [21,22,26-28,30,31,34]. Meanwhile, the other papers focus on classification tasks. As shown in Table 5, high performance is reported across both short and long prediction windows.

Some studies analyzed which variables were most important for prediction. Wu et al [17] highlighted COPD Assessment Test score, fine particulate matter (PM10), and carbon monoxide; Atzeni et al [21] emphasized symptoms in previous days and a higher frequency of exacerbations in the past 30 days; and Mohktar et al [32] identified raw FEV1 and mean SpO2 as key features. Shah et al [29] observed lower SpO2, higher respiratory rate (RR), and elevated HR during the prodromal phase, concluding that SpO2 was the most predictive feature. Overall, all studies, apart from Orchard et al [30], concluded that including all features led to better results.

Finally, models often lack explainability, which can be a fundamental element to build clinicians’ trust in the black box model. In this review, interpretability was addressed in only 7 [17,19,21,22,32-34] out of 20 studies [17-36].

#### Variables Changes in Prodromal Phases

The results of the studies related to the analysis of changes in physiological variables in prodromal phases are summarized in Table 6. Hawthorne et al [37] identified significant changes in HR and RR up to 3 days prior to exacerbations, with increases of 8.1 bpm and 2.0 breaths/minute, respectively. Coutu et al [38] found significant associations between higher EXACT-PRO (Exacerbations of Chronic Pulmonary Disease Tool–Patient-Reported Outcome) scores and reduced RR variability, step count, and sleep efficiency, suggesting that deterioration in these metrics may indicate worsening symptoms. Similarly, Zimmermann et al [39] demonstrated that variability in inspiratory reactance was significantly related to COPD Assessment Test scores.

**Table 6.** Results of included studies (variable changes in prodromal phases).

Study (year)	Outcomes	Performance metrics
Hawthorne et al [37] (2022)	Monitor changes in vital signs 3, 2, and 1 days before ECOPD^a^	HR^b^ and PA^c^ were associated with EXACT^d^ score (*P*<.001). Three days prior to exacerbation: RR^e^ increased by 2.0 (SD 0.2) breaths/min (7 of 11 cases), HR increased by 8.1 (SD 0.7) bpm (9 of 11)
Coutu et al [38] (2024)	Estimate the association between physiological variables and EXACT-PRO^f^ score	Significant unadjusted associations included RR variability (−1.45 [−2.84, −0.073] points per breath/min), daily step count (−0.56 [−0.82, −0.31] points per 1000 steps), and sleep efficiency (−0.12 [−0.20, −0.037] points per % asleep), with bracketed values representing 95% CIs.
Zimmerman et al [39] (2020)	Timing of variability in FOT^g^ measures and symptom change before AECOPD^h^	Xinsp and SDXinsp were related to mean CAT^i^ score (0.59 [1.02, 0.15], *P*=.009; 1.57 [0.65‐2.49], *P*=.001)
Al Rajeh et al [40] (2020)	Compare overnight versus daily HR and SpO2^j^ monitoring	Daily HR: max increase +7 bpm on day 1 (*P*=.007, not clinically significant). Overnight HR: +10 bpm on day 1 (*P*=.04). Composite oximetry score increased from day 7 to 0 (overnight), and on days 1 and 0 (daily): sensitivity 84.6%, PPV^k^ 91.7%
Cooper et al [41] (2020)	Identify monitored variables linked to exacerbations	Agreement between FVC^l^ drop (7-day avg 1.645 SD) and self-reported health care use (Cohen κ=0.747, *P*<.001); bronchodilator use and Anthonisen criteria (Cohen κ=0.611, *P*<.001)
Kim et al [42] (2021)	Association between PM2.5^m^ and questionnaire responses	Mean PM2.5 over 4 months was higher in patients with exacerbations (22.89, SD 5.52 g/m^3^) vs no-exacerbation patients (20.36, SD 4.63 g/m^3^), *P*<.02

a ECOPD: exacerbation of chronic obstructive pulmonary disease.

b HR: heart rate.

c PA: physical activity.

d EXACT: Exacerbations of Chronic Pulmonary Disease Tool.

e RR: respiratory rate.

f EXACT-PRO: Exacerbations of Chronic Pulmonary Disease Tool–Patient-Reported Outcome.

g FOT: Forced Oscillation Technique.

h AECOPD: acute exacerbation of chronic obstructive pulmonary disease.

i CAT: COPD Assessment Test.

j SpO2: peripheral capillary oxygen saturation.

k PPV: positive predictive value.

l FVC: forced vital capacity.

m PM2.5: particulate matter ≤2.5 µm.

Al Rajeh et al [40] compared daily versus overnight monitoring and found that overnight HR increases (+10 bpm) and composite oximetry scores provided better predictive performance for exacerbations (sensitivity 84.6%, PPV 91.7%) than daily metrics. Cooper et al [41] highlighted that significant drops in FVC aligned closely with health care use (Cohen κ=0.747), and bronchodilator use correlated with the Anthonisen criteria (Cohen κ=0.611). Finally, Kim et al [42] reported a significant association between higher ambient PM2.5 levels and increased exacerbation risk, with exposed patients showing a mean concentration of 22.89 (SD 5.52) µg/m³ compared to 20.36 (4.63) µg/m³ in those without exacerbations (*P*<.02).

Overall, these studies underline the potential of continuous monitoring of physiological and environmental variables to provide early indicators of ECOPD and support timely intervention.

## Discussion

### Principal Findings

In this section, we will discuss the key findings from the literature. We begin by examining how exacerbations are defined across studies, followed by an analysis of the most commonly monitored parameters and those shown to be discriminative for ECOPD prediction. Next, we will review the structure and limitations of predictive models, with particular emphasis on personalized approaches. Finally, we consider the devices used, identify current gaps in the literature, and outline directions for future research.

### Dataset Availability

Data are available upon reasonable request for the studies by Atzeni et al [21] and Moraza et al [34].

### Exacerbation Definition

A key issue is the lack of a universally accepted definition of COPD exacerbation, as existing classifications vary according to context, severity criteria, and data availability. This inconsistency poses challenges for ML and deep learning models, which depend on reliable and standardized outcome definitions for effective training and validation [44]. Without agreement on the condition targeted, achieving this goal becomes more difficult. As discussed in the “Results” section, this heterogeneity complicates comparisons across studies, as differing criteria can capture distinct sets of events.

To establish a unique definition, it should be based on objectively measurable variables. There is an ongoing effort in the literature to identify predictive variables for COPD exacerbations. However, there is still no consensus on which variables to use or on the time window before the exacerbation during which they show significant changes. This issue is further complicated by patient-specific thresholds and variations in physiological variables. In this context, a patient-specific model could help mitigate the situation. However, there is common agreement on the variation of HR, RR, and SpO2, with FEV1 also being very significant. Therefore, it is important to consider that the changes in these variables could help identify the onset of exacerbations.

### Variables Changes in Prodromal Phases

#### Physiological Variables

HR, RR, and SpO2 have shown promising predictive power. Moraza et al [34] identified RR, HR, and SpO2 as the most informative features. Likewise, Shah et al [29] found SpO2 to be the most predictive feature, followed by RR; while HR was the least predictive.

Al Rajeh et al [40] evaluated whether measuring changes in heart rate and oxygen saturation overnight is superior to once-daily monitoring of both variables and to assess symptom changes in facilitating earlier detection of COPD exacerbations. Concluding that COPD exacerbations are linked to measurable changes in cardiorespiratory signals, particularly HR and PEF. During the preexacerbation phase, both the mean HR and its variability increased significantly compared to the stable phase, while oxygen saturation remained consistent. Notably, overnight HR monitoring provided a clearer signal (lower variability) than once-daily measurements, enhancing the ability to detect exacerbations early. Combining HR with oxygen saturation data yielded the best performance for early detection (PPV over 90% for the detection of exacerbations), with overnight pulse oximetry outperforming once-daily monitoring.

Coutu et al [38] found that variability in RR and SpO2, rather than their absolute values, was more strongly associated with symptom fluctuations in patients with COPD. For instance, nighttime RR variability was significantly correlated with symptom scores, whereas mean RR showed no significant association in univariable analysis. Interestingly, these associations differed between individuals with persistent worsening and those who recovered, suggesting that physiological variability may serve as a more sensitive marker than single-time measurements.

Together, these findings emphasize the importance of continuous or high-resolution monitoring, particularly overnight measurements, in identifying early indications of physiological instability. However, differences in signal relevance across patient subgroups highlight the need for individualized monitoring strategies.

#### Environmental Variables

ECOPDs are influenced by a range of seasonal, environmental, and meteorological factors. Studies have shown that variables such as temperature, humidity, air pollution, and seasonal fluctuations significantly affect both the frequency and severity of ECOPDs [45,46]. Given their impact, incorporating these variables into predictive models can substantially improve model robustness and generalizability. For instance, Kim et al [42] found that patients who experienced exacerbations had been exposed to higher levels of PM2.5 in the preceding 4 months, compared to those without exacerbations.

To date, the integration of indoor environmental variables, outdoor weather conditions, and physiological variables has been explored only by Wu et al [17,19]. They reported that, compared to using clinical questionnaire data alone, incorporating lifestyle and environmental variables led to a 10% increase in accuracy and a 20% improvement in AUROC, highlighting the added predictive value of these factors.

Kolozali et al [28] found that air pollution data collected via personal air monitors were nearly as effective as peak flow measurements, reinforcing their potential predictive value. However, in Atzeni et al [21], when personal air monitor data were combined with symptom reports, SHAP (Shapley Additive Explanations) analysis indicated that symptoms and prior exacerbation history contributed more significantly to the model’s predictions. Indeed, the use of questionnaires remains a prevalent method in many studies. The main limitation of questionnaires is their reliance on active user participation, which introduces subjectivity and can intuitively lead to incomplete or missing data.

Overall, environmental variables have been shown to be discriminative and influential in exacerbation prediction, yet they remain underused in the studies reviewed. Incorporating these variables could further enhance model performance.

#### Respiratory Variables

Similarly, the extraction of respiratory features still requires portable devices rather than fully automated data collection, making the process invasive and burdensome for users. Despite their strong predictive value, features such as sound recordings, breathing patterns, and respiratory variables—including FEV1, identified as highly informative by Atzeni et al [21] and Mohktar et al [32]—and FVC drop, shown to align with exacerbation events in Cooper et al [41], present practical limitations. These methods are not well-suited for passive RM using consumer-grade devices, especially in scenarios requiring minimal user involvement.

Noninvasive monitoring methods are gaining traction, including smartphone-based algorithms capable of passively detecting cough frequency without raising privacy concerns [47]. Complementing this, Hawthorne et al [37] demonstrated that high-frequency wearable monitoring can detect physiological changes in the days leading up to an exacerbation—specifically, an average increase of 2.0 (SD 0.2) breaths/minute in RR for 7 out of 11 exacerbations, and a rise of 8.1 (SD 0.7) bpm in HR for 9 out of 11 cases.

### Studies Reporting Higher Predictive Metrics: Risk of Bias

Overall, it is important to note that several studies reporting high performance also exhibited elevated risk of bias, in particular in their analysis, participant selection domain and outcome definition. For instance, Wu et al [19] and Yin et al [22] reported AUROC values above 0.95, but relied on balanced datasets, highly selected cohorts, and did not include external validation, which are factors known to inflate performance estimates. Similarly, Nunavath et al [26] excluded moderate-risk patients and synthetically oversampled high-risk patients to balance the dataset. This does not reflect real-world prevalence and could alter results. In contrast, studies incorporating external or prospective validation, such as Van der Heijden et al [31] and Moraza et al [34], generally reported more conservative yet clinically credible performance. Furthermore, studies [18,20,23-25,31-33,36] rely on relatively small cohorts (9‐22 participants), which increases the risk of overly optimistic performance estimates. This concern is amplified by the fact that only study [31] validates its finding on an independent cohort.

Balancing of the datasets, lack of external validation, and reliance on small datasets might bias the results. As discussed in the next section, these limitations affect not only the methodological soundness of the studies but also their feasibility in real-world settings and their potential for clinical acceptance.

### Studies Reporting Higher Predictive Metrics: Limitations

One of the main limitations in comparing these studies is the lack of standardized evaluation metrics as shown in Table 7. Consistent reporting of AUROC would be beneficial, as it would highlight the model’s ability to distinguish between a random positive instance and a random negative instance, and is particularly suitable for imbalanced datasets, which is often the case for ECOPD prediction. Additionally, reporting the *F*_1_-score, which represents the harmonic mean of precision and recall, would be valuable, since accuracy alone can be misleading in imbalanced settings. Nonetheless, we compare studies reporting similar metrics, where possible.

**Table 7.** Summary of prediction results and limitations.

Study (year)	Small cohort (≤50)	Prediction window	Main results	External validation	Balanced dataset	Explainability
Wu et al [19] (2021)	No	7 days ahead	AUROC^a^=0.97, accuracy=93.6%	No	Yes	Yes (feature importance)
Merone et al [20] (2017)	Yes	Detect onset	Accuracy=98.4%, *F*_1_-score=94.0	No	No	No
Atzeni et al [21] (2022)	No	1 day ahead	AUC^b^=0.82‐0.90	No	No	Yes (SHAP^c^)
Yin et al [22] (2024)	No	1‐3 days ahead	AUROC=0.97 (k=1)	No	Yes	Yes (feature importance)
Fernandez-Granero et al [23] (2018)	Yes	~4.4-day margin	Accuracy=87.8%, *F*_1_-score=0.8	No	No	No
Wu et al [17] (2022)	No	7 days ahead	Accuracy=72.4%, *F*_1_-score=62.9%	Yes	No	Yes (feature importance and SHAP)
Kronborg et al [24] (2021)	Yes	Prodromal classification (14 days)	AUC=0.95	No	No	No
Fernandez-Granero et al [25] (2015)	Yes	~5 days ahead	Accuracy=75.8%	No	No	No
Nunavath et al [26] (2018)	No	1‐3 days ahead	Accuracy=88% (3 days)	No	No	No
Crooks et al [27] (2021)	Yes	~3 days ahead (alerts)	Alerts 3.4 days early	No	No	No
Kolozali et al [28] (2023)	No	1 day ahead	Accuracy=38.7%, *F*_1_-score=0.19	No	No	No
Shah et al [29] (2017)	No	7 days ahead	Mean AUC=0.68	No	No	No
Orchard et al [30] (2018)	No	1 day ahead	AUC=0.74	No	No	No
Van der Heijden et al [31] (2014)	Yes	1 day ahead	AUC=0.82‐0.90	Yes	No	No
Mohktar et al [32] (2015)	Yes	Next-day prodromal	Accuracy=71.8%	No	No	Yes (feature importance)
Jin et al [33] (2018)	Yes	Prodromal classification	Accuracy=~75%	No	No	Yes (feature importance)
Lin et al [18] (2020)	Yes	30-day readmission	Sensitivity=63%, precision=38%	No	No	No
Moraza et al [34] (2025)	No	3 days ahead	AUROC=0.89‐0.91	Prospective	No	Yes (SHAP)
Patel et al [35] (2021)	No	7 days ahead	Sensitivity=97.9%, NPV^d^=99.8%	No	No	No
Hsiao et al [36] (2023)	Yes	7 days ahead	Accuracy=0.94	No	No	No

a AUROC: area under the receiver operating characteristic curve.

b AUC: area under the curve.

c SHAP: Shapley Additive Explanations.

d NPV: negative predictive value.

Table 7 outlines the other limitations that were identified in our review. This includes small cohort sizes, the absence of external validation, the use of balanced datasets, limited explainability, and heterogeneity in prediction windows. Overall, these factors limit the real-world feasibility and clinical application of the proposed models.

The highest accuracy is reported in studies by Wu et al [19], Merone et al [20], and Hsiao et al [36]. However, as none of these studies included external validation on an independent cohort, the risk of overfitting remains high. This concern is particularly relevant in the study by Merone et al [20] and Hsiao et al [36], which involved very small sample sizes of 22 and 13 participants, respectively. In contrast, while the sample size in the study by Wu et al [19] is larger, a subsequent study using the same platform [17] performed external validation and reported a noticeable drop in performance. Moreover, a study by Wu et al [19] used a balanced dataset, which does not reflect real-world class distributions, thereby further limiting the reliability of the reported results. This highlights how larger cohorts and independent validation are needed for real-world feasibility. Overall, small cohorts, artificially balanced datasets, a lack of external validation, and limited model interpretability hinder the real-world applicability of current predictive models.

The highest AUC scores have been reported in studies by Yin et al [22], Kronborg et al [24], and van der Heijden et al [31]. Specifically, Yin et al [22] did not perform external validation and relied on a balanced dataset, while Kronborg et al [24] did not perform any prediction but only classified prodromal phases, and did not validate their results on an independent cohort despite the small sample size. Additionally, Yin et al [22] and van der Heijden et al [31] focus on one-day-ahead predictions, which may not provide enough time for intervention. Thus, the main limitation of these studies is their limited real-world applicability.

Due to the substantial heterogeneity across studies (Table 7), no consistent conclusion can be drawn regarding whether shorter or longer prediction windows lead to improved model performance. Nonetheless, these very short prediction windows offer limited opportunities for preventive intervention. For example, early administration of corticosteroids (oral or inhaled) has been shown to shorten the duration of exacerbations and reduce the risk of hospitalization [48]. Therefore, the primary goal should be to predict exacerbations sufficiently early to enable timely clinical intervention. Ideally, predictive models should identify the risk of exacerbation within a window of 1 to 10 days before the onset. Prediction windows that are too short (eg, 1‐2 days) may not provide adequate time for intervention, depending on the patient themselves and the severity of the exacerbation, while windows that are too long (eg, over 10 days) may incorporate excessive noise and reduce model reliability. Nevertheless, this remains uncertain, as the optimal prediction window may vary significantly between individuals. A lead time of 2 days could be sufficient in some cases, depending on patient-specific factors and the severity of the exacerbation. Thus, personalization is key.

### Personalization

Current COPD exacerbation prediction models often lack personalization. For biomedical applications, tailoring models to individual patients is crucial for improving prediction accuracy and enabling more effective interventions. In COPD-specific applications, accounting for intersubject variability and disease progression is essential, as models trained offline may struggle to adapt to changing patient states over time. In the study by Merone et al [20], parameters are optimized using data from similar patients, while Patel et al [35] incorporate a 2-week learning phase to establish a personalized baseline. While both models achieve good results, they do not provide a comparison to population-level models. In contrast, Kolozali et al [28] include this comparison, demonstrating improved accuracy and *F*_1_-score for the personalized model over the general one. Finally, Atzeni et al [21] attempted to cluster patients, identifying 2 distinct groups. Cluster 1 includes patients with milder COPD, characterized by higher median PEF values and mild-to-moderate disease severity. In contrast, Cluster 2 consists of patients with more severe COPD who reported going out fewer days per week. Despite these differences, models trained on the entire dataset and evaluated separately on each cluster performed similarly to cluster-specific models.

### Devices

One research question of this review examines the types of wearable IoT devices used for the RM of COPD. In the last decade, wearable devices, IoT sensors, and AI-enabled technologies have increasingly been recognized as invaluable instruments, allowing continuous, noninvasive, and real-time monitoring of diseased conditions and health [49]. Since COPD is chronic, it is essential to gather data continuously to detect trends and track disease progression. This is where wearable devices and home sensors come in handy to enable continuous RPM. This approach ensures that data collection happens passively, without requiring active involvement from patients, who are often older adults. Nevertheless, 2 key points emerged following the article screening.

First, wearable devices are primarily used in studies that analyze changes in vital parameters during prodromal periods [37-42]. Thanks to the high granularity of the collected data, subtle deviations from baseline can be highlighted before, during, and after exacerbation phases. However, these high-frequency data are often leveraged for modeling purposes rather than for early prediction.

Second, although recent RM approaches increasingly incorporate wearable devices that enable near-continuous remote collection and transmission of physiological data, research integrating these data streams into predictive models remains limited. So far, research combining RM and continuous data acquisition via IoT devices is still in its early stages. The few studies that integrate wearable devices with prediction models—such as [17-19,21,28,36]—typically rely on summary statistics, thereby losing the granular insights offered by high-frequency continuous data.

The current literature is limited by a lack of standardization in both device usage and the reporting of sampling frequency. Therefore, there is no evidence yet to draw conclusions on which is the most suitable device, or even to determine whether continuous data improves outcomes with respect to sampled data. Additionally, the frequent omission of raw sampling frequency represents a critical methodological gap. Reporting sampling frequencies should therefore be established as a reporting standard in future RPM research.

Finally, although wearable devices enable out-of-home monitoring, this review focused on in-home monitoring. This focus reflects the available published literature rather than the application of exclusion criteria related to out-of-home monitoring. Indeed, no search criteria excluded out-of-home monitoring. This outcome likely mirrors the typical daily routines of many patients with COPD rather than a lack of interest in out-of-home monitoring. COPD predominantly affects older patients with reduced mobility and advanced disease stages, often affected by various comorbidities, such as diabetes, cardiovascular diseases, and/or cancer. As a result, in-home monitoring enables more continuous, reliable, and clinically meaningful data collection while minimizing patient burden and adherence issues. That said, we fully acknowledge that out-of-home monitoring remains an important and underexplored area, particularly for earlier disease stages or more active patients.

### Models and Future Directions

As discussed above, most studies rely on ML techniques that require substantial human effort for feature engineering and often fail to capture temporal patterns, which may be significant on their own. van der Heijden et al [31] showed that dynamic Bayesian networks, learned from small, sparse clinical time series using structural expectation-maximization and bootstrapping, can accurately predict COPD exacerbations and are suitable for use in chronic disease management tools. Similarly, Nunavath et al [26] used a Long Short-Term Memory model to predict the health conditions of patients with COPD one day ahead with an accuracy of 84.12%. Thus, proving that neural networks, especially Long Short-Term Memory, show strong potential for remote COPD monitoring and decision support. However, both approaches rely on once-daily measurements, limiting their ability to capture high-resolution temporal dynamics. This limitation suggests that alternative pattern mining and time-series analysis techniques should be explored to assess whether finer-grained temporal data can further improve prediction accuracy.

It is also crucial to note that time-series analysis is particularly critical in small-data clinical settings. However, when compared with traditional ML techniques, time-series approaches may present important drawbacks, including limited explainability and an increased risk of overfitting when applied to small datasets. Additional challenges may include sensitivity to noise and missing data, which are frequent when data come from wearable sensors. Despite these limitations, when carefully designed and validated, time-series approaches hold significant promise for improving exacerbation prediction models.

In traditional ML pipelines, feature engineering and selection are crucial, and they often rely on domain knowledge. However, COPD symptoms and progression may have complex patterns that are difficult to handcraft into features. Therefore, it is hard to identify quantitative features to improve the prediction’s outcome. To address these limitations, recent research has explored self-supervised learning (SSL) techniques, which not only can automatically extract latent features from biomedical signals, but also leverage a massive amount of unlabeled data, thus avoiding manual labeling. In SSL frameworks, models are trained using pretext tasks such as masked prediction, contrastive learning, or next-step prediction, and the learned representations can later be fine-tuned on smaller labeled datasets for downstream tasks. This paradigm has been shown to improve generalizability and robustness, making SSL a promising approach for COPD exacerbation forecasting.

Self-supervised learning has been successfully applied in many fields, such as natural language processing, computer vision, speech recognition, and robotics. In medical research, computer vision is the most investigated area [50]. Most existing COPD exacerbation studies focus on supervised learning with labeled datasets of vital signs, symptoms, and activity levels. For instance, studies typically involve random forests, survival analysis, or neural networks trained on labeled datasets to predict exacerbations by tracking vital parameters such as respiratory rate, oxygen saturation, and HR. However, as discussed, labeling exacerbations is not an easy task due to the conflicting and subjective definitions of the event. In this framework, SSL relies on unlabeled data or, more precisely, it does require labels but not human labeling, since pseudolabels are created during the pretext tasks. In pretext tasks, pseudolabels are used for representations that are generated automatically by considering the attributes of the dataset [51]. Still, even if not labeled, the large-scale dataset should contain lots of ECOPD events.

Some recent studies explore unsupervised or self-supervised approaches in medical anomaly detection, suggesting that these methods could provide useful features to improve early detection in COPD if adapted effectively for health care data structures. Several recent studies are indeed exploring self-supervised approaches for detecting and predicting COPD exacerbations or related anomalies, particularly focusing on imaging data and anomaly detection methods. Almeida et al [52] developed a self-supervised anomaly detection framework that identifies abnormal lung regions in patients with COPD, using computed tomography imaging and creating an “anomaly map” for lung regions. This framework allows for early detection of variations in lung health and highlights phenotypic manifestations such as airway disease and emphysema. This approach leverages self-supervised learning to capture data complexity without requiring detailed labels, making it practical for diverse, heterogeneous COPD presentations. The cOOpD study (derived from COPD and out-of-distribution [OOD] concepts) [53] poses COPD classification as an anomaly detection problem, applying contrastive learning to computed tomography scans of healthy lungs to establish a baseline of “normal” lung characteristics. The study leverages this baseline to identify deviations in patients with COPD, which is especially helpful in the absence of labeled exacerbation events. The study demonstrated promising applications in phenotyping COPD and predicting declines in lung function by detecting early abnormal patterns in lung images.

With some effort, SSL techniques can be applied to time series signals, such as those extracted from wearable devices, to achieve early prediction of ECOPD without the need for labels. For instance, recent work by Google [54] introduced a Large Sensor Model with adaptive and inherited masking, a novel SSL framework designed to learn robust representations directly from incomplete and heterogeneous wearable sensor data. Although not developed specifically for COPD, such a framework could be pretrained on large-scale unlabeled wearable data and subsequently fine-tuned, in downstream tasks, for ECOPD prediction. This approach may be particularly advantageous in real-world clinical settings, where sensor data are often missing or irregular and annotated exacerbation events are limited.

### Comparison With Prior Work

Similar systematic reviews have already been published, but substantial differences will now be discussed.

Sanchez-Morillo et al [55] published a systematic review of existing algorithms for the detection of exacerbations and clinical decision-making in the context of COPD and asthma. The article collects papers published between 2005 and 2015. First, the focus is not exclusively on COPD; second, it does not cover RM and IoT sensors, and furthermore, it is now outdated, not including the influence of environmental factors on ECOPD.

Al Rajeh and Hurst [56] conducted a systematic review on the effectiveness of monitoring physiological variables to predict ECOPDs, with a search updated until 2016. Out of 15 studies, 9 studies showed a reduction in SpO2 prior to exacerbation onset, 3 studies for peak flow, and 2 studies for respiratory rate reported a significant variation prior to or at exacerbation onset [56]. But exploring the significance of changes in variables in prodromal periods does not cover the predictive frameworks.

Finally, Glyde et al [57] published a dual narrative synthesis. One part focuses on RCTs evaluating RPM interventions aimed at treating or improving acute exacerbations of chronic obstructive pulmonary disease (AECOPD) and asthma, while the other examines ML models combined with RPM to predict AECOPD, with most studies assessing accuracy using retrospective data. In contrast, our review broadens the scope by including details on sensing technologies, real-world applicability, and future directions. We also present studies investigating changes in physiological variables during the prodromal phases of AECOPD, and focusing only on COPD.

### Limitations

The main limitation of the present work is that data heterogeneity prevented a formal meta-analysis. Moreover, although the literature search was comprehensive, only a few studies met the inclusion criteria, making it difficult to draw statistically robust conclusions.

### Conclusions

#### RQ1: Physiological and Environmental Variables, Data Collection, and Device Use

The review of studies modeling changes in physiological parameters during the prodromal phases of AECOPD highlights the importance of HR and oxygen saturation. Variations over time in these parameters appeared more discriminative than their absolute values. Environmental exposures were also found to have a significant impact on the incidence of exacerbations; however, these data remain underused. We therefore suggest that multimodal data integration represents a promising strategy for further improving ECOPD prediction.

Recently, there has been a shift toward using parameters measurable through wearable devices, reflecting the growing emphasis on continuous and noninvasive monitoring in COPD management. However, there is currently no standardization in device types, data acquisition protocols, and reporting of sampling frequency, limiting comparability across studies. Furthermore, despite the use of continuous monitoring, most studies rely on aggregated features and therefore do not fully exploit the granularity of the collected data.

#### RQ2: ML Approaches, Metrics, and Exacerbation Definitions

Numerous models have been developed for the prediction of ECOPD, with some demonstrating high performance. However, the absence of standardized output metrics hinders meaningful comparison across studies. In addition, key aspects such as external validation, real-time deployment, and the choice of prediction windows remain underexplored. The lack of a unique definition of ECOPD complicates model training and evaluation, as detection is often subjective and symptom-based. While personalization and the use of high-granularity data show potential for earlier detection, these approaches require more evidence to establish their effectiveness.

#### RQ3: Synthesis, Limitations, and Future Directions

Bringing these findings together, extracting meaningful patterns from high-frequency physiological and environmental data using IoT sensors could enable automated RM of COPD. Integrating time-series analysis with personalized baselines, along with ensuring external validation in independent cohorts and model interpretability, are critical next steps. Indeed, the lack of external validation and interpretability remains a major barrier to the application of AI models in clinical practice. Furthermore, many studies rely on small cohorts and, in some cases, artificially balanced datasets, which further limits their generalizability to real-world settings.

If implemented within a reasonable timeframe before symptom escalation, such models could allow timely interventions, potentially mitigating exacerbations and improving quality of life for patients with COPD, while also reducing the associated economic burden.

## Supplementary material

10.2196/84814 Multimedia Appendix 1 Search Strings for each database.

10.2196/84814 Checklist 1 PRISMA checklist.

## Abbreviations

AECOPD: acute exacerbations of chronic obstructive pulmonary disease
AI: artificial intelligence
AUC: area under the curve
AUROC: area under the receiver operating characteristic curve
COPD: chronic obstructive pulmonary disease
ECOPD: exacerbations of chronic obstructive pulmonary disease
ER: emergency room
EXACT-PRO: Exacerbations of Chronic Pulmonary Disease Tool–Patient-Reported Outcome
FEV1: forced expiratory volume in 1 second
FOT: Forced Oscillation Technique
FVC: forced vital capacity
GOLD: Global Initiative for Chronic Obstructive Lung Disease
HR: heart rate
IoT: Internet of Things
MeSH: Medical Subject Headings
ML: machine learning
OOD: out-of-distribution
PEF: peak expiratory flow
PM2.5: particulate matter ≤2.5 µm
PPV: positive predictive value
PRISMA: Preferred Reporting Items for Systematic Reviews and Meta-Analyses
PROBAST: Prediction Model Risk of Bias Assessment Tool
RCT: randomized controlled trial
RM: remote monitoring
RPM: remote patient monitoring
RR: respiratory rate
SHAP: Shapley Additive Explanations
SpO2: peripheral capillary oxygen saturation
SSL: self-supervised learning
