# Supplementary material for: AI and Internet of Things for Chronic Obstructive Pulmonary Disease Remote Monitoring: Systematic Review of Exacerbation Prediction and Key Physiological Variables
Source: JMIR Med Inform. 2026 May 6;14:e84814. doi: 10.2196/84814 (PMC13148758; doi:10.2196/84814)
Supplement: Multimedia Appendix 1 [file medinform-v14-e84814-s001.docx]

| DATABASE | QUERY | RESULTS |
| --- | --- | --- |
| PubMed | ("Wearable Electronic Devices"[MeSH] OR "wearable*"[tiab] OR "sensor*"[tiab] OR "Remote Monitoring"[tiab]  OR "Remote Patient Monitoring"[tiab] OR "Home Monitoring"[tiab] OR "Telemonitoring"[tiab] OR "Digital Health"[tiab]  OR "Passive Monitoring"[tiab] OR "mHealth"[tiab] OR "eHealth"[tiab] OR "Physiological Monitoring"[tiab]  OR "biosensor*"[tiab] OR "smartwatch*"[tiab] OR "oximeter"[tiab] OR "spirometer"[tiab] OR "heart rate monitor"[tiab]  OR "wearable technology"[tiab] OR "physiological signal"[tiab] OR "ECG"[tiab] OR "PPG"[tiab])  AND ("Chronic Obstructive Pulmonary Disease"[MeSH] OR "COPD"[tiab] OR "Pulmonary Disease, Chronic Obstructive"[MeSH]  OR "AECOPD"[tiab] OR "ECOPD"[tiab] OR "Chronic Respiratory Disease"[tiab] OR "Lung Function Decline"[tiab])  AND ("Machine Learning"[MeSH] OR "Artificial Intelligence"[MeSH] OR "Predictive Modeling"[tiab]  OR "Prediction"[tiab] OR "Risk Assessment"[tiab] OR "Early Detection"[tiab] OR "Forecasting"[tiab]  OR "Clustering"[tiab] OR "Neural Networks"[tiab] OR "Feature Selection"[tiab] OR "Anomaly Detection"[tiab]) | 125 |

| DATABASE | QUERY | RESULTS |
| --- | --- | --- |
| IEEE Xplore | ("wearable*" OR "sensor*" OR "Remote Monitoring" OR "Remote Patient Monitoring" OR "Home Monitoring" OR "Telemonitoring"  OR "Digital Health" OR "Passive Monitoring" OR "mHealth" OR "eHealth" OR "Physiological Monitoring" OR "biosensor*" OR "smartwatch*" OR "oximeter" OR "spirometer" OR "heart rate monitor" OR "wearable technology" OR "physiological signal" OR "ECG" OR "PPG")  AND ("Chronic Obstructive Pulmonary Disease" OR "COPD" OR "AECOPD" OR "ECOPD" OR "Obstructive Lung Disease*"  OR "Chronic Respiratory Disease" OR "Lung Function Decline")  AND ("Machine Learning" OR "Artificial Intelligence" OR "Deep Learning" OR "Supervised Learning" OR "Unsupervised Learning" OR "Predictive Modeling" OR "Prediction" OR "Risk Assessment" OR "Early Detection"  OR "Forecasting" OR "Clustering" OR "Neural Networks" OR "Feature Selection" OR "Anomaly Detection") | 155 |
| DATABASE | **QUERY** | **RESULTS** |
| Scopus | TITLE-ABS-KEY ( "wearable*" OR "sensor" OR "inertial*" OR "IMU*" OR "  ("wearable*" OR "sensor*" OR "Remote Monitoring" OR "Remote Patient Monitoring" OR "Home Monitoring" OR "Telemonitoring"  OR "Digital Health" OR "Passive Monitoring" OR "mHealth" OR "eHealth" OR "Physiological Monitoring" OR "biosensor*" OR "smartwatch*" OR "oximeter" OR "spirometer" OR "heart rate monitor" OR "wearable technology" OR "physiological signal" OR "ECG" OR "PPG")  AND TITLE-ABS-KEY ("Chronic Obstructive Pulmonary Disease" OR "COPD" OR "AECOPD" OR "ECOPD" OR "Obstructive Lung Diseases"  OR "Chronic Respiratory Disease" OR "Lung Function Decline")  AND TITLE-ABS-KEY ("Machine Learning" OR "Artificial Intelligence" OR "Deep Learning" OR "Supervised Learning" OR "Unsupervised Learning" OR "Predictive Modeling" OR "Prediction" OR "Risk Assessment" OR "Early Detection" OR "Forecasting" OR "Clustering" OR "Neural Networks" OR "Feature Selection" OR "Anomaly Detection") | 922 |

| DATABASE | QUERY | RESULTS |
| --- | --- | --- |
| EMBASE | ('wearable electronic device'/exp OR 'wearable*':ti,ab,kw OR 'sensor*':ti,ab,kw OR 'inertial*':ti,ab,kw OR 'IMU*':ti,ab,kw  OR 'remote monitoring':ti,ab,kw OR 'remote patient monitoring':ti,ab,kw OR 'home monitoring':ti,ab,kw OR 'telemonitoring':ti,ab,kw OR 'digital health':ti,ab,kw OR 'passive monitoring':ti,ab,kw OR 'mHealth':ti,ab,kw  OR 'eHealth':ti,ab,kw OR 'physiological monitoring':ti,ab,kw OR 'biosensor*':ti,ab,kw OR 'smartwatch*':ti,ab,kw OR 'oximeter':ti,ab,kw OR 'spirometer':ti,ab,kw OR 'heart rate monitor':ti,ab,kw OR 'wearable technology':ti,ab,kw OR 'physiological signal':ti,ab,kw OR 'ECG':ti,ab,kw OR 'PPG':ti,ab,kw)  AND ('chronic obstructive pulmonary disease'/exp OR 'COPD':ti,ab,kw OR 'AECOPD':ti,ab,kw OR 'ECOPD':ti,ab,kw  OR 'obstructive lung disease':ti,ab,kw OR 'chronic respiratory disease':ti,ab,kw OR 'lung function decline':ti,ab,kw)  AND ('machine learning'/exp OR 'artificial intelligence'/exp OR 'deep learning':ti,ab,kw OR 'supervised learning':ti,ab,kw  OR 'unsupervised learning':ti,ab,kw OR 'predictive modeling':ti,ab,kw OR 'prediction':ti,ab,kw OR 'risk assessment':ti,ab,kw  OR 'early detection':ti,ab,kw OR 'forecasting':ti,ab,kw OR 'clustering':ti,ab,kw OR 'neural networks':ti,ab,kw  OR 'feature selection':ti,ab,kw OR 'anomaly detection':ti,ab,kw) | 481 |
